# Identification and Assessment of Outcome Measurement Instruments in Cauda Equina Syndrome: A Systematic Review

**DOI:** 10.1177/21925682241227916

**Published:** 2024-01-17

**Authors:** George E. Richardson, Christopher P. Millward, James W. Mitchell, Simon Clark, Martin Wilby, Anthony G. Marson, Paula R. Williamson, Nisaharan Srikandarajah

**Affiliations:** 1 School of Medicine, 4591 University of Liverpool, Liverpool, UK; 2 Institute of Systems, Molecular, and Integrative Biology, 4591 University of Liverpool, Liverpool, UK; 3 Department of Neurosurgery, The Walton Centre NHS Foundation Trust, Liverpool, UK; 4 Department of Neurology, The Walton Centre NHS Foundation Trust, Liverpool, UK; 5 Institute of Population Health, 4591 University of Liverpool, Liverpool, UK; 6 Institute of Translational Medicine, 4591 University of Liverpool, Liverpool, UK

**Keywords:** cauda equina syndrome, core outcome set, outcome measurement instrument

## Abstract

**Study Design:**

This was a systematic review of surgically managed Cauda Equina Syndrome (CES) Outcome Measurement Instruments (OMI).

**Objective:**

A core outcome set (COS) defines agreed outcomes which should be reported as a minimum in any research study for a specific condition. This study identified OMIs used in the wider CES literature and compare these to the established CESCOS.

**Methods:**

To identify measurement methods and instruments in the CES surgical outcome evidence base, a systematic review was performed. Medline, Embase and CINAHL plus databases were queried. In addition, a secondary search for validation studies of measurement instruments in CES was undertaken. Identified studies from this search were subject to the COSMIN risk of bias assessment.

**Results:**

In total, 112 studies were identified investigating surgical outcomes for CES. The majority (80%, n = 90) of these OMI studies were retrospective in nature and only 55% (n = 62) utilised a measurement method or instrument. The remaining 50 studies used study specific definitions for surgical outcomes defined within their methods. Of the 59 measurement instruments identified, 60% (n = 38 instruments) were patient reported outcome measures. Only one validated instrument was identified, which was a patient reported outcome measure. The validated instrument was not used in any study identified in the initial search (to identify measurement instruments).

**Conclusions:**

This review highlights the wide heterogeneity of measurement instruments used in surgically managed CES research. Subsequently, there is need for consensus agreement on which instrument or instruments should be used to measure each core outcome for CES surgical outcomes.

## Introduction

Cauda equina syndrome (CES) results from compression of the nerve roots emerging below the termination of the spinal cord.^1^ Cauda equina syndrome occurs due to prolapsed intervertebral disc in 45% of cases but can result from any compressive pathology.^2^ There is no unanimous clinical definition of CES, however common features include perineal sensory disturbance, pain, genitourinary dysfunction, and lower limb motor deficits.^1,3^ Cauda equina syndrome is a clinically important issue as the physiological, occupational and social ramifications of its sequelae can be devastating for patients.^4^ As such, it is a true neurological emergency and requires urgent surgical intervention when compressive pathology is present.^4^

A core outcome set (COS) defines an agreed minimum set of outcomes that should be reported by research for a specific health condition or health area. They enable attempts to provide an overview of existing evidence including systematic review and meta-analyses, reduce reporting bias, and ensure investigation of clinically important outcomes.^5^ A cauda equina syndrome core outcome set (CESCOS) has previously been defined^6^ with an international group of healthcare professionals and patients using consensus methodology. The CESCOS identified the minimum standard outcomes of surgically managed CES which research studies should be reporting.^6^ Non-surgically managed CES was not included in CESCOS and as such was not a focus of the present study.^6^ The core outcomes from CESCOS are outlined in the methods section of this article.

Outcome measurement instruments (OMI) are tools and frameworks used within research to measure a pre-specified outcome, for example the Landriel Ibañez classification for neurosurgical post-operative morbidity.^7^ Outcome measurement instrument’s can include questionnaires, laboratory measurements, and specific clinical definitions. The COnsensus-based Standards for the selection of health Measurement INstruments (COSMIN) initiative is formed by a multi-disciplinary team of researchers with expertise in the field of OMI’s.^8^ The initiative has a number of key aims, including the standardisation of research outcomes by the development of COSs.^8^ COnsensus-based Standards for the selection of health Measurement INstrument provides guidance on performing reviews of OMIs, including Patient Reported Outcome Measures (PROMs) and Clinician Reported Outcome Measures (ClinROMs).^8^ Guidance also exists for identification of OMI’s for the defined outcomes in a COS.^9^ Only one third of core outcome set developers proceed to the next step, the development of a Core Outcome Measurement Set (COMS).^10^ The lack of COMS is a known barrier to the uptake of COS within clinical trials.^11^ Currently, there is no consensus agreement available on which measurement instruments should be used to measure the defined core outcomes in CES.^6^ Cauda Equina Syndrome core OMIs are needed to further improve the comparability of similar studies in this area, facilitate meta-analysis and reduce research waste.^12^

### Rationale and Objectives

The purpose of this study is to perform a systematic review of commonly used PROMs and ClinROMs in the CES evidence base. Additionally, this study also identifies and evaluates studies seeking to develop or validate research instruments for use in CES.

## Methods

This is a systematic review of the OMI’s used in the reporting of clinical studies for CES. The methodological approach to identifying research measurement instruments has previously been defined and a similar method was undertaken to pre-existing literature.^8,13^ No patient consent or ethical approval was required for this study.

### Inclusion and Exclusion Criteria

For the initial database search (see below), studies were eligible for inclusion if patients had a diagnosis of CES and had undergone surgery for the condition. All forms of clinical study were eligible for inclusion, however pre-clinical and animal studies were excluded. A cohort of equal to or greater than 5 patients was required, and paediatric studies were excluded. For the validation studies search (see below), the same criteria were applied with one addition. To satisfy inclusion criteria, the studies must have contained evidence related to one of the COSMIN checklist criteria (e.g. validity, reliability, interpretability or feasibility). The full inclusion criteria are outlined in Table 1.

**Table 1.** Inclusion Criteria for Eligible Studies.

Criteria for the Identification of Outcome Measurement Instruments	Criteria for the Identification of Measurement Instruments Validation Studies
Diagnosis of CES	Diagnosis of CES
Patients have undergone surgery for the pathology causing CES	Patients have undergone surgery for the pathology causing CES
Randomized controlled trials, non-randomized controlled trials, prospective and retrospective cohort studies, qualitative and case series	Contained evidence pertaining to one of the COSMIN checklist criteria (e.g. validity, reliability, interpretability or feasibility)
English language	Primary research
Five or more patients	English language
Published between 1990 and 2022	Five or more patients
Adult patients aged 16 years and above	Published between 1990 and 2022
	Adult patients aged 16 years and above

### Database Search and Data Extraction to Identify Outcome Measurement Instruments

To identify studies containing OMIs, 3 databases were searched. Search strategies for Ovid (Medline), Ovid (Embase), and CINAHL Plus were developed. The full searches are outlined in Supplementary Materials 1. Studies published within the inclusion time-period (between January 1, 1990 and April 30, 2022) were extracted. This time period was split into 2 discrete searches. First, a historic search described in Srikandarajah et al provided studies from January 1, 1990 to September 30, 2016.^14^ In Srikandarajah et al. the authors provide a review of reported outcomes for surgically managed CES patients, within the CES evidence base. The search strategy results from this study formed the basis of the initial search in the present study, as the evidence base was the same, and only the OMI’s used required extracting from the prior results. Additionally, this search was updated using a qualitative study filter as the historic search had excluded studies of this type. Updating the historic search whilst applying the qualitative filter allowed the original screening results to be used despite the difference in inclusion/exclusion criteria. This search was then updated using an identical strategy to cover January 1, 2015 to the April 30, 2022. An overlap of 1 year was included to reduce the chances of erroneously missed studies. Articles were single screened by 2 authors (G.E.R. and N.S.). Following screening, approximately 10% of all screened records were checked by the senior authors (N.S., M.W. and S.C.).

Data extraction was performed using a pre-defined proforma. Again, approximately 10% of the extracted studies were checked by a senior author (N.S.) to ensure concordance, after which extraction proceeded without further checking. Baseline details were extracted for all records including, first author, publishing journal, year published, study type, and whether it involved patient data collection using a measurement instrument. Study type was defined as either, Randomised Control Trial (RCT), Non-Randomised Control Trial (N-RCT), prospective cohort, retrospective cohort, case series, qualitative or cross sectional. For studies containing measurement instruments, the name of the OMI and the outcome it measured in the study were recorded. Outcome categories were mapped to the “Core Outcomes” defined for use in CES and are illustrated in Table 2.^6^

**Table 2.** Core Outcomes Defined for Use in CES.^6^

Outcome Number	Outcome
CO1	Incontinence of urine
CO2	Urinary retention
CO3	Sensation of bladder fullness
CO4	Faecal incontinence
CO5	Physical ability to have sexual intercourse
CO6	Perineal sensation
CO7	Sensation in genitals
CO8	Leg muscle strength
CO9	Pain due to abnormal sensation or non-painful stimulus
CO10	Complications (including death)
CO11	Global quality of life
CO12	Occupational role functioning
CO13	Social role functioning
CO14	Ability to do daily activities
CO15	Mobility and walking
CO16	Low mood and depression

### Database Search and Data Extraction for Instrument Validation Studies

To identify studies investigating the validity of measurement instruments in CES, a separate search was conducted. The search covered the period between September 1, 1990 and May 30, 2022. The full search strategy is provided in Supplementary Materials 1. To identify validation studies, the sensitive PubMed search filter was employed from Terwee et al.^15^ This search filter was developed to identify studies on PubMed that look specifically at the measurement properties of measurement instruments, rather than the OMI’s themselves.^15^ No other tool is available to serve this purpose.^15^ The search was only conducted on PubMed since the filter was developed specifically for this platform. Screening of articles was performed by one author (G.E.R.). Approximately 10% of all screened records were checked with a senior author (N.S.).

### Risk of Bias Assessment for Included Measurement Instrument Validation Studies

To evaluate the risk of bias for measurement instrument validation studies, the 2018 COSMIN risk of bias checklist was used.^8,16^ For each component of the checklist, studies are scored on a four-point scale from “very good”, “adequate”, “doubtful” to “inadequate”.^16^ Evaluation of content validity was the primary step when evaluating instruments and was evaluated according to published guidance.^17^ Studies with inadequate content validity were not considered for further evaluation.^8^ Next, evaluation of internal structure was performed by considering structural validity, internal consistency, and cross-cultural validity/measurement invariance.^8^ Evaluation of the remaining measurement properties was then performed. These include, reliability, measurement error, criterion validity, hypotheses testing for construct validity, and responsiveness.^8^ Risk of bias assessment was undertaken independently by 2 authors (G.E.R. and N.S.). Difference in scoring were discussed and any disagreements were settled between the authors.

## Results

### Studies Containing Outcome Instruments

**Figure 1.**
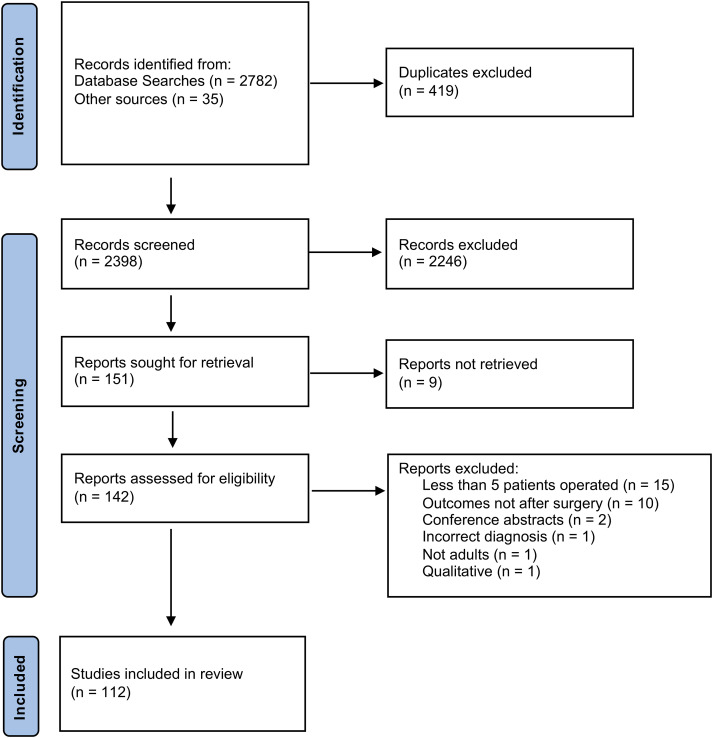
PRISMA flow chart for combined instrument identification search.

A total of 112 studies investigating surgical outcomes for CES were included. The PRISMA flowchart for the combined instrument search is demonstrated in Figure 1.

**Figure 2.**
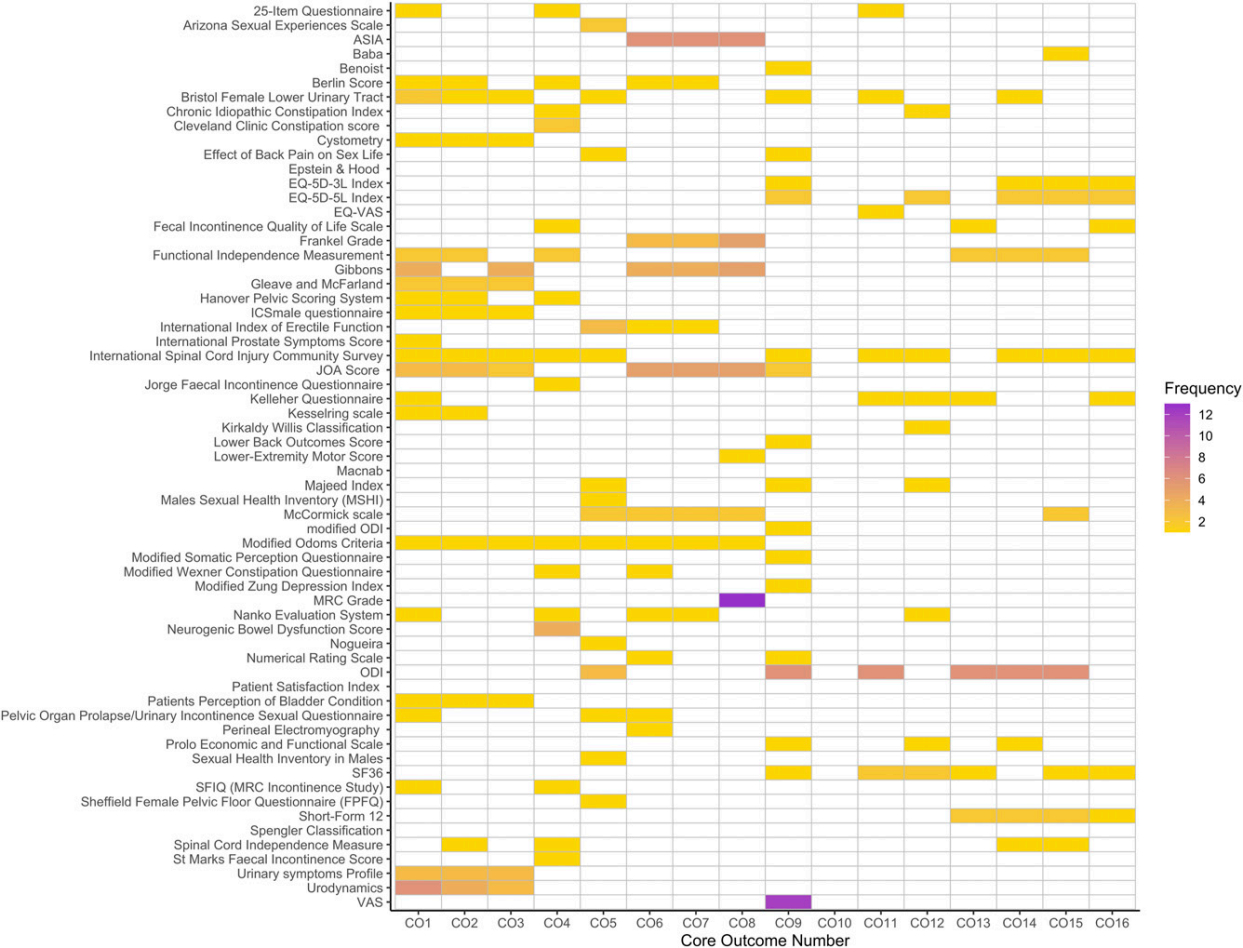
Heatmap demonstrating measurement instrument use frequency by respective core outcome. Core outcomes numbers are defined in Table 2.

The full list of studies and their OMI is included in Supplementary document 2. The types of studies included are outlined in Table 3. From these studies, 62 (55%) contained some form of measurement instrument. A total of 63 unique OMI’s were identified from the included studies, of which 38 (60%) were PROMs and 25 (40%) were physician administered instruments. The 2 most frequently used instruments were the Medical Research Council (MRC) grade (measuring motor function – CO8) and the Visual Analogue Scale (VAS) (measuring pain – CO9), which were included in 13 and 12 studies respectively. The OMI which measured the most core outcomes was the International Spinal Cord Injury Community Survey, which covered 11 of the core outcomes (see Figure 2 for more details). There were no OMIs identified in the search which measured morbidity and mortality (core outcome 10). The vast majority of OMIs were applied in the same way (used to measure the same core outcomes) between different studies. The frequency of measurement instrument use mapped to the relevant core outcomes is illustrated in Figure 2.

**Table 3.** Frequencies and Percentages of Study Types Included in the Systematic Review.

Study Design	Frequency (%)
Retrospective cohort	90 (80.4)
Prospective cohort	16 (14.3)
Qualitative	3 (2.7)
Cross sectional	2 (1.8)
N-RCT	1 (.9)

### Measurement Instrument Validation Studies

**Figure 3.**
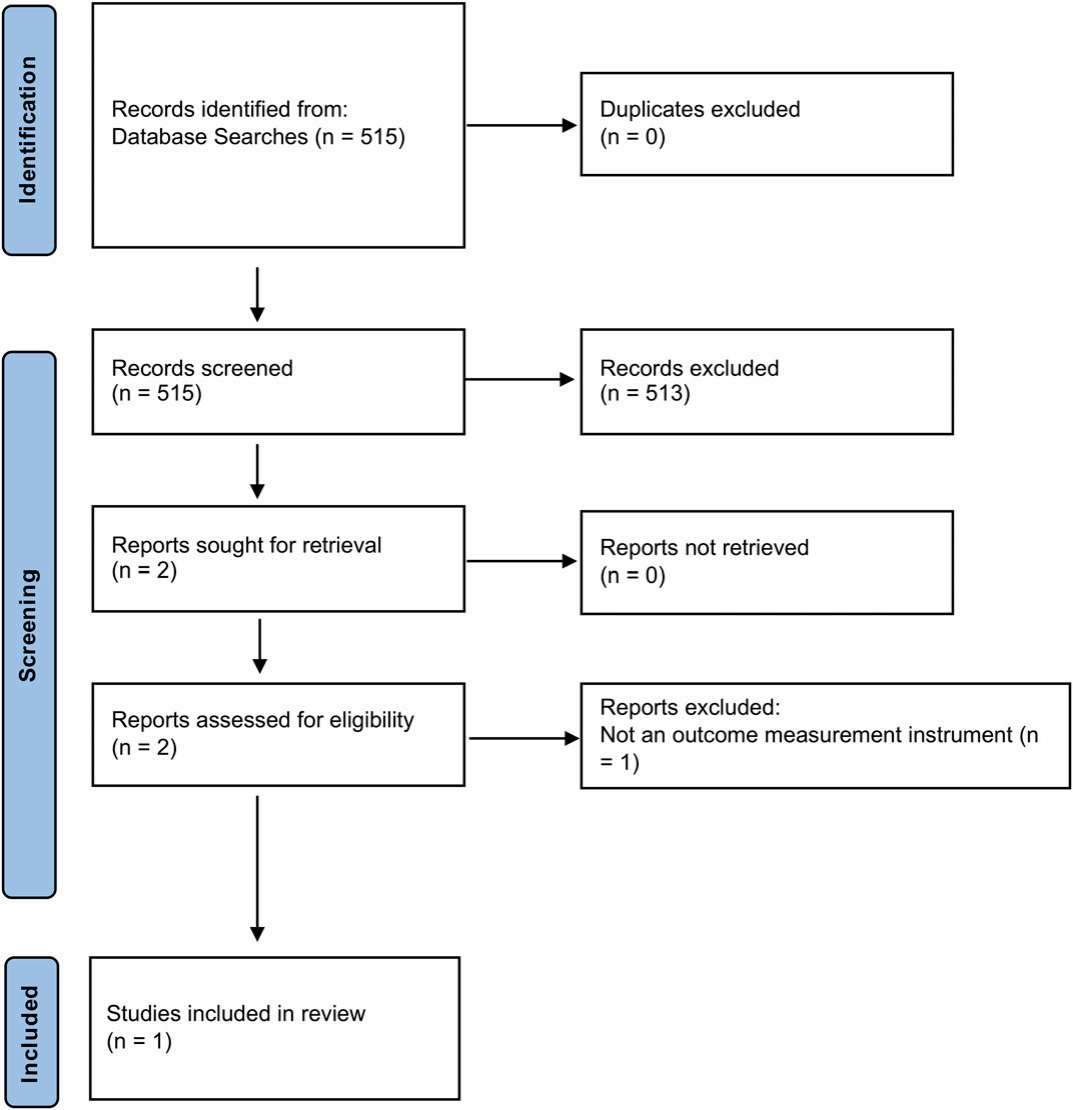
PRISMA flow chart for instrument validation study search.

One study validating a measurement instrument in CES was identified. The PRISMA flowchart for the validation study search is shown in Figure 3. A search of the COSMIN database for the OMI’s identified in the initial search (Figure 1) identified that 13 of the instruments had been included in prior COSMIN reviews. These 13 OMIs were included in reviews relevant to spinal cord injury, however there were no OMIs included in reviews relevant to CES.

The study identified did not exclusively validate an instrument in a population of CES patients and instead formed part of a composite cohort. In the study, the authors sought to validate a foreign language version of an instrument in patients with multiple sclerosis and spinal cord injury, the latter of which contained a smaller cohort of CES patients.^18^ The instrument was not included in any of the studies from the initial search strategy (Figure 1).

In Noordhoff et al the authors sought to validate the Multiple Sclerosis Intimacy and Sexuality Questionnaire (MSISQ-15) in a cohort of Dutch language speaking participants.^18^ Six Cauda Equina patients were included alongside forty-three spinal cord injury patients as one composite cohort. Participants were asked to complete the MSISQ-15 questionnaire at their initial enrolment and complete it a second time 2 weeks later at home. The questionnaire set included the MSISQ-15, the EQ visual analogue scale (EQ-VAS), Pelvic Organ Prolapse/Urinary Incontinence Sexual Questionnaire (PISQ-12), and International Index of Erectile Function (IIEF-15). Results were compared to a reference cohort recruited from a single general practitioner’s patient group. Content validity was deemed to be adequate during the linguistic validation process and found the translated version of the questionnaire to be understandable. Internal consistency of the MSISQ-15 was deemed to be good, with a Cronbach’s alpha of >.8 for test-retest in all cohort groups. Of note, within the spinal cord injury cohort (containing Cauda Equina patients), the primary domain of the MSISQ-15 demonstrated only moderate internal consistency with a Cronbach’s alpha of .53 and .55 (for test and retest, respectively). Reliability analysis demonstrated adequate (>.7) intraclass correlation coefficients for the MSISQ-15 and the 3 domains in all participant cohorts. Pearson’s correlation coefficients demonstrated significant relationships between the MISISQ-15 and PISQ-12/IIEF-15 in female/male cohorts respectively.

### COSMIN Assessment of Methodology

The study by Noordhoff et al was assessed according to the COSMIN standards for evaluating the quality of content validity in studies of PROMs.^18^ The results of the COSMIN analysis are demonstrated in Table 4.

**Table 4.** Results From COSMIN Analysis of Validation Study.

Instrument and Location	Multiple Sclerosis Intimacy and Sexuality Questionnaire (MSISQ-15). In The Netherlands, conducted in Dutch		
Population	Total study population n = 152 (53 with MS, 49 with spinal cord disorders [including 6 with CES], and 50 control group). Adult patients.		
Content validity	Asking patients	Relevance	D
		Comprehensiveness	D
		Comprehensibility	D
	Asking experts	Relevance	D
		Comprehensiveness	D
Structural validity			N
Internal consistency			V
Cross-cultural validity			I
Reliability			V
Measurement error			A
Criterion validity			A
Construct validity		Convergent validity	V
		Known groups validity	A
Responsiveness		Comparison with Gold standard	A
		Comparison with other instruments	V
		Comparison between subgroups	A
		Comparison before and after intervention	A

*Abbreviations*: V = very good, A = adequate, D = doubtful, I = inadequate, N = not applicable.

## Discussion

### Key Findings

This review identified a total of 63 unique measurement instruments within the current CES evidence base. The most frequently used measurement instruments were the MRC grade of muscle power and the VAS of pain. One study was identified seeking to develop or validate instruments for use in CES populations. This study was not specifically validating for use in CES alone and the OMI was not used within the wider CES evidence base (e.g. identified during the initial CES OMI search).

### Outcome Measurement Instrument Validation and Heterogeneity

The number of validation studies identified in this review was very low, even in comparison to similarly rare diseases. For example, COSMIN database reviews have identified 23 measurement instruments or methods validated for use in Huntington’s disease.^19-22^ Both conditions have a similar prevalence, and the disparity between number of available instruments for CES should trigger a call for further work to validate instrument for use in ongoing research. Moreover, of the validation studies that were highlighted by this review, none were specifically focused on cohorts of patients with CES and the instrument being developed or validated were not implemented within the wider CES evidence base. As previously stated, consensus methodology should be used to decide if identified measurement instruments are suitable to quantify the core outcomes. Suitable instruments would then require validation for use within surgically managed CES, as highlighted above by the lack of currently validated instrument. The pragmatic short-term solution to this is to adopt multiple pre-existing measurement instruments, a combination of which would address all the defined core outcomes in CESCOS. Such a combination of instruments could then be validated to ensure their adequacy. The only foreseeable drawback to this approach is that no instrument identified for the purpose of quantifying morbidity. This could possibly be rectified by seeking outside the CES specific literature and validating a pre-existing complications grading system, such as the Therapies-Disability-Neurology grade.^23^

Within this review a large number of unique OMI’s were identified. The outcomes measured by these instruments varied greatly, as did the degree to which they were implemented. The MRC grade and VAS were the most frequently used measurement instruments.^24,25^ Their high frequency of use is likely related to their ubiquitous nature in other topic areas, the simplicity with which they can be applied, and their low burden of implementation. Many of the other instruments identified were used only a single time. The result is reduced comparability between similar CES studies and limited opportunities for meaningful pooled meta-analyses. Consensus methodology should be utilised to obtain an agreement among key stakeholders on how each core outcome should be measured, and if the identified measurement instruments are appropriate to do so.

For complications (core outcome 10), no CES specific instrument was identified within any of the current evidence base. This is because studies presented data on morbidity and mortality in its raw format. For example, limb weakness following surgery would be reported as mentioned, rather than within an established complication grading system (such as the aforementioned Therapies-Disability-Neurology grade).^23^ This raises the issue of whether all core outcomes require a specific measurement instrument, for example mortality can be easily defined clinically, however such omissions from COMS would still require consensus agreement. The instrument identified with the highest utility in this regard was the International Spinal Cord Injury Community Survey (ISCICS), addressing a total of eleven core outcomes defined in the CESCOS, however this was used in only one study of CES patients and no studies were found validating this for use specifically in cauda equina. The ISCICS itself was developed with the aim of investigating the lived experiences of spinal cord injury patients on an international scale, of which CES was included.^26^ The ISCICS was an amalgamation of existing measurement instruments, including the SF-36, Model Disability Survey, and the General Self-Efficacy Scale.^26^ The world health organisation’s international classification of functioning, disability and health (ICF) was used as a framework against which the outcomes of interest were selected.^26^ The higher degree of utility related to the core outcomes defined relates to breadth of outcomes included within the ICF.^27^

This study represents a single step in the ongoing work to improve the reporting quality of surgically managed CES. The next step would be consensus agreement on which OMIs are appropriate for each outcome in the CESCOS. Then, using the recommended CESCOS and the appropriate OMIs for them, a study can be designed to adequately investigate the short- and long-term outcomes for patients with CES after surgery.

### Limitations

Studies in this review were subject only to single author review. This was felt to be appropriate as similar methodology was employed for the previous study on which the original search strategy was based.^14^ It is possible that measurement instruments identified in the initial search may have been validated in a broader population still of relevance to CES but were not included as the validation search focused solely on CES patient cohorts.

## Conclusion

This review highlights the current measurement instruments utilised in the surgically managed CES literature, within the context of the defined core outcomes. Further work is needed to validate adequate OMI for the measurement of these outcomes specifically in CES populations. As with the development of COSs, a consensus approach involving key stakeholders should take place to decide which measurement instruments best achieve the requirements of ongoing surgically managed CES research.

## Supplemental Material

Supplemental Material - Identification and Assessment of Outcome Measurement Instruments in Cauda Equina Syndrome: A Systematic Review Supplemental Material for Identification and Assessment of Outcome Measurement Instruments in Cauda Equina Syndrome: A Systematic Review by George E. Richardson, Christopher P. Millward, James W. Mitchell, Simon Clark, Martin Wilby, Anthony G. Marson, Paula R. Williamson, and Nisaharan Srikandarajah in Global Spine Journal

Supplemental Material - Identification and Assessment of Outcome Measurement Instruments in Cauda Equina Syndrome: A Systematic Review Supplemental Material for Identification and Assessment of Outcome Measurement Instruments in Cauda Equina Syndrome: A Systematic Review by George E. Richardson, Christopher P. Millward, James W. Mitchell, Simon Clark, Martin Wilby, Anthony G. Marson, Paula R. Williamson, and Nisaharan Srikandarajah in Global Spine Journal

## Data Availability

A list of included studies has been provided in the supplementary data. The search strategies have also been provided to allow for reproduction of study methodology.

## References

[1] Woodfield J Hoeritzauer I Jamjoom AAB , et al. Presentation, management, and outcomes of cauda equina syndrome up to one year after surgery, using clinician and participant reporting: a multi-centre prospective cohort study. Lancet Reg Health Europe. 2023;24:100545. doi:10.1016/j.lanepe.2022.100545 36426378 PMC9678980

[2] Korse NS Pijpers JA van Zwet E Elzevier HW Vleggeert-Lankamp CLA . Cauda equina syndrome: presentation, outcome, and predictors with focus on micturition, defecation, and sexual dysfunction. Eur Spine J. 2017;26(3):894-904. doi:10.1007/s00586-017-4943-8 28102451

[3] Fraser S Roberts L Murphy E . Cauda equina syndrome: a literature review of its definition and clinical presentation. Arch Phys Med Rehabil. 2009;90(11):1964-1968. doi:10.1016/j.apmr.2009.03.021 19887225

[4] Gardner A Gardner E Morley T . Cauda equina syndrome: a review of the current clinical and medico-legal position. Eur Spine J. 2011;20(5):690-697. doi:10.1007/s00586-010-1668-3 21193933 PMC3082683

[5] Williamson P Altman D Blazeby J Clarke M Gargon E . Driving up the quality and relevance of research through the use of agreed core outcomes. J Health Serv Res Policy. 2012;17(1):1-2. doi:10.1258/jhsrp.2011.011131 22294719

[6] Srikandarajah N Noble A Clark S , et al. Cauda Equina Syndrome Core Outcome Set (CESCOS): an international patient and healthcare professional consensus for research studies. PLoS One. 2020;15(1):e0225907. doi:10.1371/journal.pone.0225907 31923259 PMC6953762

[7] Landriel Ibañez FA Hem S Ajler P , et al. A new classification of complications in neurosurgery. World Neurosurg. 2011;75(5-6):709-715; discussion 604-611. doi:10.1016/j.wneu.2010.11.010 21704941

[8] Prinsen CAC Mokkink LB Bouter LM , et al. COSMIN guideline for systematic reviews of patient-reported outcome measures. Qual Life Res. 2018;27(5):1147-1157. doi:10.1007/s11136-018-1798-3 29435801 PMC5891568

[9] Prinsen CAC Vohra S Rose MR , et al. How to select outcome measurement instruments for outcomes included in a “core outcome set” – a practical guideline. Trials. 2016;17(1):449. doi:10.1186/s13063-016-1555-2 27618914 PMC5020549

[10] Gorst SL Prinsen CAC Salcher-Konrad M Matvienko-Sikar K Williamson PR Terwee CB . Methods used in the selection of instruments for outcomes included in core outcome sets have improved since the publication of the COSMIN/COMET guideline. J Clin Epidemiol. 2020;125:64-75. doi:10.1016/j.jclinepi.2020.05.021 32470621

[11] Hughes KL Williamson PR Young B . In-depth qualitative interviews identified barriers and facilitators that influenced chief investigators’ use of core outcome sets in randomised controlled trials. J Clin Epidemiol. 2022;144:111-120. doi:10.1016/j.jclinepi.2021.12.004 34896233 PMC9094758

[12] Webbe J Sinha I Gale C . Core outcome sets. Arch Dis Child Educ Pract Ed. 2018;103(3):163. doi:10.1136/archdischild-2016-312117 28667046

[13] Lu JD Hobbs MM Huang WW Ortega-Loayza AG Alavi A . Identification and evaluation of outcome measurement instruments in pyoderma gangrenosum: a systematic review. Br J Dermatol. 2020;183(5):821-828. doi:10.1111/bjd.19027 32159849

[14] Srikandarajah N Wilby M Clark S Noble A Williamson P Marson T . Outcomes reported after surgery for cauda equina syndrome: a systematic literature review. Spine (Phila Pa 1976). 2018;43(17):E1005-E1013. doi:10.1097/brs.0000000000002605 29432394 PMC6104724

[15] Terwee CB Jansma EP Riphagen II de Vet HC . Development of a methodological PubMed search filter for finding studies on measurement properties of measurement instruments. Qual Life Res. 2009;18(8):1115-1123. doi:10.1007/s11136-009-9528-5 19711195 PMC2744791

[16] Mokkink LB de Vet HCW Prinsen CAC , et al. COSMIN risk of bias checklist for systematic reviews of patient-reported outcome measures. Qual Life Res. 2018;27(5):1171-1179. doi:10.1007/s11136-017-1765-4 29260445 PMC5891552

[17] Terwee CB Prinsen CA Chiarotto A , et al. COSMIN Methodology for Assessing the Content Validity of PROMs – User Manual; 2022. https://www.cosmin.nl/ (Accessed June 21, 2022).

[18] Noordhoff TC Scheepe JR t Hoen LA Sluis TAR Blok BFM . The multiple sclerosis intimacy and sexuality questionnaire (MSISQ-15): validation of the Dutch version in patients with multiple sclerosis and spinal cord injury. Neurourol Urodyn. 2018;37(8):2867-2874. doi:10.1002/nau.23804 30168628

[19] Mestre TA Bachoud-Lévi AC Marinus J , et al. Rating scales for cognition in Huntington’s disease: critique and recommendations. Mov Disord. 2018;33(2):187-195. doi:10.1002/mds.27227 29278291 PMC10080408

[20] Mestre TA Busse M Davis AM , et al. Rating scales and performance-based measures for assessment of functional ability in Huntington’s disease: critique and recommendations. Mov Disord Clin Pract. 2018;5(4):361-372. doi:10.1002/mdc3.12617 30363510 PMC6174516

[21] Mestre TA Carlozzi NE Ho AK , et al. Quality of life in Huntington’s disease: critique and recommendations for measures assessing patient health-related quality of life and caregiver quality of life. Mov Disord. 2018;33(5):742-749. doi:10.1002/mds.27317 29570848

[22] Mestre TA Forjaz MJ Mahlknecht P , et al. Rating scales for motor symptoms and signs in Huntington’s disease: critique and recommendations. Mov Disord Clin Pract. 2018;5(2):111-117. doi:10.1002/mdc3.12571 30363393 PMC6174417

[23] Terrapon APR Zattra CM Voglis S , et al. Adverse events in neurosurgery: the novel therapy-disability-neurology grade. Neurosurgery. 2021;89(2):236-245. doi:10.1093/neuros/nyab121 33887774

[24] Paternostro-Sluga T Grim-Stieger M Posch M , et al. Reliability and validity of the Medical Research Council (MRC) scale and a modified scale for testing muscle strength in patients with radial palsy. J Rehabil Med. 2008;40(8):665-671. doi:10.2340/16501977-0235 19020701

[25] Langley GB Sheppeard H . The visual analogue scale: its use in pain measurement. Rheumatol Int. 1985;5(4):145-148. doi:10.1007/bf00541514 4048757

[26] Gross-Hemmi MH Post MWM Ehrmann C , et al. Study protocol of the international spinal cord injury (InSCI) community survey. Am J Phys Med Rehabil. 2017;96(2):S23-S34. doi:10.1097/phm.0000000000000647 28059876

[27] Vargus-Adams JN Majnemer A . International classification of functioning, disability and health (ICF) as a framework for change: revolutionizing rehabilitation. J Child Neurol. 2014;29(8):1030-1035. doi:10.1177/0883073814533595 24850572

