# Supplementary material for: Identification and Assessment of Outcome Measurement Instruments in Cauda Equina Syndrome: A Systematic Review
Source: Global Spine J. 2024 Jan 17;14(6):1818–26. doi: 10.1177/21925682241227916 (PMC11268298; doi:10.1177/21925682241227916)
Supplement: Supplemental Material - Identification and Assessment of Outcome Measurement Instruments in Cauda Equina Syndrome: A Systematic Review [file sj-pdf-1-gsj-10.1177_21925682241227916.pdf]

Original Search

Ovid Medline 01/01/1990- 30/09/2016

Search #	Search term	Results
1	exp Polyradiculopathy/	2485
2	(case report or abstract).mp. [mp=title, abstract, original title, name of substance word, subject heading word, keyword heading word, protocol supplementary concept word, rare disease supplementary concept word, unique identifier, synonyms]	1980773
3	Animals/	6104266
4	1 not 2	2119
5	4 not 3	1996
6	limit 5 to english language	1253
7	limit 6 to yr=”1990 -2016”	650

Ovid Embase 01/01/1990- 30/09/2016

Search #	Search term	Results
1	cauda equina syndrome.af.	2580
2	(case report or abstract).af.	13016786
3	animal.af.	5369655
4	(cauda equina syndrome not (case report or abstract)).af.	1191
5	(cauda equina syndrome not (case report or abstract) not animal).af.	1116
6	limit 5 to english language [Limit not valid in Your Journals@Ovid; records were retained]	993
7	Limit 6 to yr=”1990 –2016”	949

CINAHL Plus 01/01/1990- 30/09/2016

Search #	Search term	Search Options	Results
1	Cauda equina syndrome		330
2	Cauda equina syndrome NOT (case report or abstract)		252
3	Cauda equina syndrome NOT (case report or abstract) NOT (animal)		246
4	Cauda equina syndrome NOT (case report or abstract) NOT (animal)	**Narrow by Language:** - english	241
5	Cauda equina syndrome NOT (case report or abstract) NOT (animal)	**Narrow by Language:** - english<br>**Limiters** - Publication Year: 1990-2016	239

Present Day Search

Medline – 01/01/2015 - 30/04/2022

Search #	Search term	Results
1	exp Polyradiculopathy/	2580
2	(case report or abstract).mp. [mp=title, abstract, original title, name of substance word, subject heading word, keyword heading word, protocol supplementary concept word, rare disease supplementary concept word, unique identifier, synonyms]	1993540
3	Animals/	7117051
4	1 not 2	2211
5	4 not 3	2086
6	limit 5 to english language	1330
7	limit 6 to yr= 2015 -Current	143

Embase – 01/01/2015 - 30/04/2022

Search #	Search term	Results
1	cauda equina syndrome.af.	3447
2	(case report or abstract).af.	13453228
3	animal.af.	6183785
4	(cauda equina syndrome not (case report or abstract)).af.	1629
5	(cauda equina syndrome not (case report or abstract) not animal).af.	1536
6	limit 5 to english language [Limit not valid in Your Journals@Ovid; records were retained]	1409
7	Limit 6 to yr= 2015 – Current	573

CINAHL plus – 01/01/2015 - 30/04/22

Search #	Search term	Search Options	Results
1	Cauda equina syndrome		668
2	Cauda equina syndrome NOT (case report or abstract)		511
3	Cauda equina syndrome NOT (case report or abstract) NOT (animal)		478
4	Cauda equina syndrome NOT (case report or abstract) NOT (animal)	**Narrow by Language:** - english	472
5	Cauda equina syndrome NOT (case report or abstract) NOT (animal)	**Narrow by Language:** - english<br>**Limiters** - Publication Year: 2015-Current	220

Qualitative Re-run

Medline Ovid - 01/01/1990- 30/09/2016

Search #	Search term	Results
1	exp Polyradiculopathy/	2578
2	(case report or abstract).mp. [mp=title, abstract, original title, name of substance word, subject heading word, keyword heading word, protocol supplementary concept word, rare disease supplementary concept word, unique identifier, synonyms]	1992390
3	Animals/	7112769
4	1 not 2	2209
5	4 not 3	2084
6	limit 5 to english language	1328
7	limit 6 to yr="1990 - 2017"	681
8	((("semi-structured" or semistructured or unstructured or informal or "in-depth" or indepth or "face-to-face" or structured or guide) adj2 (interview* or discussion* or questionnaire*)) or (focus group* or qualitative or ethnograph* or fieldwork or "field work" or "key informant")).tw,kw. or interviews as topic/ or focus groups/ or narration/ or qualitative research/	465195
9	7 and 8	3

Embase Ovid - 01/01/1990- 30/09/2016

Search #	Search term	Results
1	cauda equina syndrome.af.	3445
2	(case report or abstract).af.	13437039
3	animal.af.	6171207
4	(cauda equina syndrome not (case report or abstract)).af.	1627
5	(cauda equina syndrome not (case report or abstract) not animal).af.	1534
6	limit 5 to english language [Limit not valid in Your Journals@Ovid; records were retained]	1407
7	Limit 6 to yr=1990 – 2017	984
8	(("semi-structured" or semistructured or unstructured or informal or "in-depth" or indepth or "face-to-face" or structured or guide) adj3 (interview* or discussion* or questionnaire*)).ti,ab. or (focus group* or qualitative or ethnograph* or fieldwork or "field work" or "key informant").tw,kw. or qualitative research/	533230
9	7 and 8	4

CINAHL Plus - 01/01/1990- 30/09/2016

Search #	Search term	Search Options	Results
1	Cauda equina syndrome		660
2	Cauda equina syndrome NOT (case report or abstract)		509
3	Cauda equina syndrome NOT (case report or abstract) NOT (animal)		483
4	Cauda equina syndrome NOT (case report or abstract) NOT (animal)	**Narrow by Language:** - english	477
5	Cauda equina syndrome NOT (case report or abstract) NOT (animal)	**Narrow by Language:** - english<br>**Limiters** - Publication Year: 1990-2016	337
6	Cauda equina syndrome NOT (case report or abstract) NOT (animal)	**Narrow by Language:** - english<br>**Limiters** - Publication Year: 1990-2016<br>**Expert Filters -** Clinical Queries: Qualitative - Best Balance	1

Medline + Embase :
https://extranet.santecom.qc.ca/wiki/!biblio3s/doku.php?id=concepts:recherche-qualitative

CINHAL Plus: Expert filters on CINHAL Plus search
Qualitative – best balance

Inclusion Criteria:

**TABLE 1. Inclusion Criteria for the review**
Diagnosis of CES
Patients have undergone surgery for the pathology causing CES
Randomized controlled trials, nonrandomized controlled trials, prospective and retrospective cohort studies, qualitative and case series
Human studies
English language
Five or more patients
Published between 1990 to 2022
Adult patients aged 16 years and above

Validation searches

PubMed – 01/01/1990 - 30/05/2022

Search #	Search term	Results
1	Cauda Equina Syndrome	1,956
2	(instrumentation[sh] OR methods[sh] OR Validation Studies[pt] OR Comparative Study[pt] OR "psychometrics"[MeSH] OR psychometr*[tiab] OR clinimetr*[tw] OR clinometr*[tw] OR "outcome assessment (health care)"[MeSH] OR outcome assessment[tiab] OR outcome measure*[tw] OR "observer variation"[MeSH] OR observer variation[tiab] OR "Health Status Indicators"[Mesh] OR "reproducibility of results"[MeSH] OR reproducib*[tiab] OR "discriminant analysis"[MeSH] OR reliab*[tiab] OR unreliab*[tiab] OR valid*[tiab] OR coefficient[tiab] OR homogeneity[tiab] OR homogeneous[tiab] OR "internal consistency"[tiab] OR (cronbach*[tiab] AND (alpha[tiab] OR alphas[tiab])) OR (item[tiab] AND (correlation*[tiab] OR selection*[tiab] OR reduction*[tiab])) OR agreement[tiab] OR precision[tiab] OR imprecision[tiab] OR "precise values"[tiab] OR test–retest[tiab] OR (test[tiab] AND retest[tiab]) OR (reliab*[tiab] AND (test[tiab] OR retest[tiab])) OR stability[tiab] OR interrater[tiab] OR inter-rater[tiab] OR intrarater[tiab] OR intra-rater[tiab] OR intertester[tiab] OR inter-tester[tiab] OR intratester[tiab] OR intra-tester[tiab] OR interobserver[tiab] OR inter-observer[tiab] OR intraobserver[tiab] OR intra-observer[tiab] OR intertechnician[tiab] OR inter-technician[tiab] OR intratechnician[tiab] OR intra-technician[tiab] OR interexaminer[tiab] OR inter-examiner[tiab] OR intraexaminer[tiab] OR intra-examiner[tiab] OR interassay[tiab] OR inter-assay[tiab] OR intraassay[tiab] OR intra-assay[tiab] OR interindividual[tiab] OR inter-individual[tiab] OR intraindividual[tiab] OR intra-individual[tiab] OR interparticipant[tiab] OR inter-participant[tiab] OR intraparticipant[tiab] OR intra-participant[tiab] OR kappa[tiab] OR kappa's[tiab] OR kappas[tiab] OR repeatab*[tiab] OR ((replicab*[tiab] OR repeated[tiab]) AND (measure[tiab] OR measures[tiab] OR findings[tiab] OR result[tiab] OR results[tiab] OR test[tiab] OR tests[tiab])) OR generaliza*[tiab] OR generalisa*[tiab] OR concordance[tiab] OR (intraclass[tiab] AND correlation*[tiab]) OR discriminative[tiab] OR "known group"[tiab] OR factor analysis[tiab] OR factor analyses[tiab] OR dimension*[tiab] OR subscale*[tiab] OR (multitrait[tiab] AND scaling[tiab] AND (analysis[tiab] OR analyses[tiab])) OR item discriminant[tiab] OR interscale correlation*[tiab] OR error[tiab] OR errors[tiab] OR "individual variability"[tiab] OR (variability[tiab] AND (analysis[tiab] OR values[tiab])) OR (uncertainty[tiab] AND (measurement[tiab] OR measuring[tiab])) OR "standard error of measurement"[tiab] OR sensitiv*[tiab] OR responsive*[tiab] OR ((minimal[tiab] OR minimally[tiab] OR clinical[tiab] OR clinically[tiab]) AND	9,362,998

	(important[tiab] OR significant[tiab] OR detectable[tiab]) AND (change[tiab] OR difference[tiab])) OR (small*[tiab] AND (real[tiab] OR detectable[tiab]) AND (change[tiab] OR difference[tiab])) OR meaningful change[tiab] OR "ceiling effect"[tiab] OR "floor effect"[tiab] OR "Item response model"[tiab] OR IRT[tiab] OR Rasch[tiab] OR "Differential item functioning"[tiab] OR DIF[tiab] OR "computer adaptive testing"[tiab] OR "item bank"[tiab] OR "cross-cultural equivalence"[tiab])	
3	#1 AND #2	526
4	3 Limited 1990-2022	515

Validation inclusion criteria

**TABLE 1. Inclusion Criteria for the review**
Diagnosis of CES
Patients have undergone surgery for the pathology causing CES
Contained evidence pertaining to one of the COSMIN checklist criteria (e.g. validity, reliability, interpretability or feasibility)
Primary research (e.g. Trial, cohort, case series)
Human studies
English language
Five or more patients
Published between 1990 to 2022
Adult patients aged 16 years and above
